# Protein kinase Cδ promotes proliferation and induces malignant transformation in skeletal muscle

**DOI:** 10.1111/jcmm.12452

**Published:** 2014-10-06

**Authors:** Gabriella Czifra, Attila Szöllősi, Zsuzsanna Nagy, Miklós Boros, István Juhász, Andrea Kiss, Ferenc Erdődi, Tamás Szabó, Ilona Kovács, Miklós Török, László Kovács, Peter M Blumberg, Tamás Bíró

**Affiliations:** a DE-MTA “Lendület” Cellular Physiology Research Group, Department of Physiology, Medical Faculty, University of Debrecen, Research Center for Molecular MedicineDebrecen, Hungary; b Department of Dermatology, Medical Faculty, University of Debrecen, Research Center for Molecular MedicineDebrecen, Hungary; c Department of Medical Chemistry, Medical Faculty, University of Debrecen, Research Center for Molecular MedicineDebrecen, Hungary; d Department of Pediatrics, Medical Faculty, University of Debrecen, Research Center for Molecular MedicineDebrecen, Hungary; e Department of Pathology, Gyula Kenézy HospitalDebrecen, Hungary; f Laboratory of Cancer Biology and Genetics Center for Cancer Research, National Cancer InstituteBethesda, MD, USA

**Keywords:** Skeletal muscle, C2C12 myoblasts, rhabdomyosarcoma, protein kinase C, nPKCδ, PKC isoenzymes, recombinant overexpression, proliferation, differentiation, tumourigenesis

## Abstract

In this paper, we investigated the isoform-specific roles of certain protein kinase C (PKC) isoforms in the regulation of skeletal muscle growth. Here, we provide the first intriguing functional evidence that nPKCδ (originally described as an inhibitor of proliferation in various cells types) is a key player in promoting both *in vitro* and *in vivo* skeletal muscle growth. Recombinant overexpression of a constitutively active nPKCδ in C2C12 myoblast increased proliferation and inhibited differentiation. Conversely, overexpression of kinase-negative mutant of nPKCδ (DN-nPKCδ) markedly inhibited cell growth. Moreover, overexpression of nPKCδ also stimulated *in vivo* tumour growth and induced malignant transformation in immunodeficient (SCID) mice whereas that of DN-nPKCδ suppressed tumour formation. The role of nPKCδ in the formation of rhabdomyosarcoma was also investigated where recombinant overexpression of nPKCδ in human rhabdomyosarcoma RD cells also increased cell proliferation and enhanced tumour formation in mouse xenografts. The other isoforms investigated (PKCα, β, ε) exerted only minor (mostly growth-inhibitory) effects in skeletal muscle cells. Collectively, our data introduce nPKCδ as a novel growth-promoting molecule in skeletal muscles and invite further trials to exploit its therapeutic potential in the treatment of skeletal muscle malignancies.

## Introduction

The protein kinase C (PKC) system is a central intracellular signalling pathway regulating various cellular processes such as proliferation, differentiation, apoptosis and tumourigenesis 1–4. Up to date, at least 11 PKC isoenzymes were identified which can be classified to the calcium- and phorbol ester-dependent ‘conventional’ (PKCα, βI, βII and γ; cPKCs); the calcium-independent ‘novel’ (PKCδ, ε, η and θ; nPKCs); the calcium- and phorbol ester-independent ‘atypical’ (PKCζ, and λ/ι; aPKCs); and PKD groups. These isoforms isozyme-specifically and very often differentially regulate the given cellular mechanism 3,5,6. Furthermore, not only may some PKC isoforms be active whereas others not for a given response but different PKC isoforms may have antagonistic effects on the same cellular event 7–9.

Various PKC isoforms were shown to control certain cellular functions in skeletal muscles as well. For example, nPKCθ were implicated in mediating the complex effect of insulin to control muscle homoeostasis 10, whereas cPKCα and nPKCδ were shown to participate in the effect of tumour necrosis factor-α to inhibit insulin signalling 11,12. In addition, nPKCδ and aPKCζ were found to positively regulate glucose and monocarboxylate transport 13–15 while aPKCζ and λ were documented to play a role in the regulation of exercise-related changes in metabolic and gene-regulatory responses of human skeletal muscle 16.

We, however, possess extremely limited information about the isoform-specific involvement of the PKCs in the regulation of physiological and pathological *in vitro* and *in vivo* growth of skeletal muscle cells 17,18. cPKCα was introduced as a central promoter of cellular growth of cultured avian myoblasts 19,20 while nPKCθ was suggested to promote differentiation of mouse 21 and human 22 skeletal muscles. PKC isoforms are suggested to function as oncogenes in rhabdomyosarcoma (RMS), the most common and lethal skeletal muscle sarcomas in children. Indeed, the phosphorylation levels of cPKCα, nPKCδ, nPKCθ and aPKCs are up-regulated in alveolar and embryonal RMS as well 23.

We have previously shown 24 that nPKCδ – which isoform was previously suggested to inhibit proliferation, induces apoptosis and/or promotes differentiation 9 – plays a pivotal and exclusive role in mediating the *in vitro* growth-promoting effect of insulin-like growth factor-I (IGF-I) both in human skeletal muscle cultures and in the mouse C2C12 skeletal muscle myoblast cell line (which is very often used to model growth and differentiation of this tissue 25,26).

Therefore, as a continuation of the above study, in the present work – using combined molecular biology (recombinant overexpression), pharmacology (inhibitors), as well as *in vivo* assay (tumourigenesis in SCID mice) – our goal was to further dissect the role of nPKCδ in the regulation of *in vitro* and, or further importance, *in vivo* growth of the cells. In addition, we also intended to define the specific roles of several other PKC isoforms in skeletal muscle growth. We report here for the first time that nPKCδ functions as a novel signalling molecule to promote *in vitro* and *in vivo* cell growth as well as to induce malignant transformation of skeletal muscle myoblasts.

## Materials and methods

### Antibodies for Western blotting

All primary antibodies against PKC isoforms were developed in rabbits and were shown to react specifically with the given PKC isoforms 9,24,27. Anti-PKCα, β, and ε were from Sigma-Aldrich (St. Louis, MO, USA), whereas anti-PKCδ was from Santa Cruz BioTech (Santa Cruz, CA, USA). Specificities of anti-PKC antibodies were also tested by applying isoform-specific blocking peptides, which blocked the immunostaining in all cases 9. Monoclonal mouse antibody against the intermediate filament protein desmin was from DAKO (Glostrup, Denmark). p44/42 MAP kinase (ERK 1/2) and phospho-p44/42 MAP kinase (phospho-ERK 1/2) antibodies were from Cell Signaling Technology (Beverly, MA, USA). In addition, monoclonal rabbit β-actin antibody (Sigma-Aldrich) was employed as internal control.

### Generation of PKC constructs

Protein kinase C constructs were engineered as described previously 9,24,27–31. Briefly, the cDNA sequences of PKCα, β, δ, and ε and of the kinase (dominant)-negative (DN-nPKCδ) mutant of nPKCδ were subcloned into a metallothionein promoter-driven eukaryotic expression vector (MTH) 32. The vector sequence encodes a C-terminal PKCε-derived 12 amino acid tag (εMTH) and attaches it to the end of the PKC proteins. As we previously described 29,30, this epitope tag does not affect the functional properties of the given isoform.

### Cell culture and transfection of cells

The C2C12 myoblasts (obtained from the American Type Culture Collection, ATCC No. CRL-1772) were cultured in DMEM (Sigma-Aldrich) supplemented with 15% (v/v) foetal calf serum (Sigma-Aldrich), 2 mM l-glutamine (Sigma-Aldrich), 50 U/ml penicillin, 50 μg/ml streptomycin, 1.25 μg/ml Fungizone (both from PAA Laboratories GmbH, Austria). Human RMS-derived RD cells (obtained from the American Type Culture Collection, ATCC No. CCL-136) were maintained in DMEM (Sigma-Aldrich) supplemented with 10% (v/v) foetal bovine serum (Invitrogen, Paisley, UK), 2 mM Glutamine (Sigma-Aldrich), 50 U/ml penicillin and 50 μg/ml streptomycin (both from TEVA). Medium was changed every other day and cells were sub-cultured at 80% confluence at 37°C in a humidified atmosphere with 5% CO_2_.

**Fig 1:**
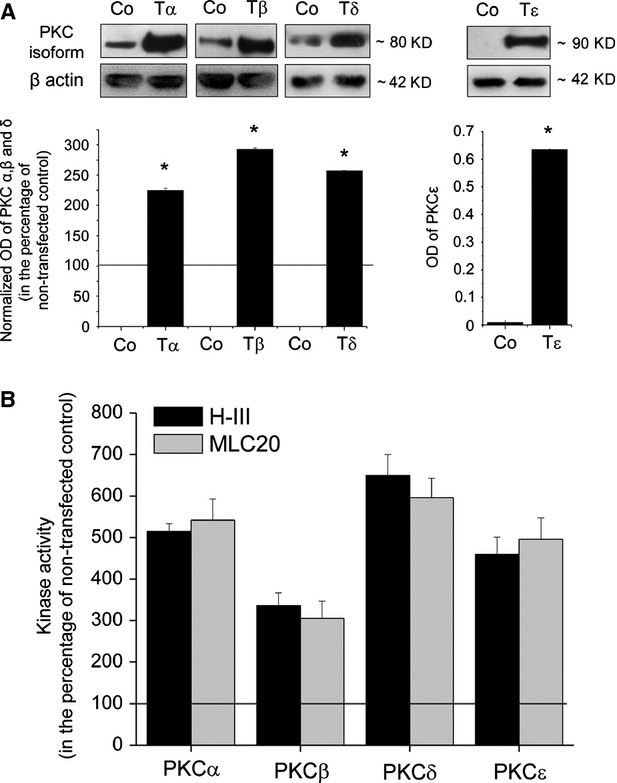
Overexpression of certain PKC isoforms in C2C12 myoblasts. Stable transfectants of C2C12 cells (Tα, Tβ, Tδ, Tɛ) overexpressing the different PKC isoforms or the empty pɛMTH vector (Control, Co) were harvested, similar amounts of proteins were subjected to SDS-PAGE, blotting was performed and the membranes were probed with isoform-specific antibodies to PKC isoformes (A). To assess equal loading, membranes were re-probed with β-actin antibody (β actin). The amounts of PKC isoforms were quantitated by densitometry (optical density; OD), and expressed as the percentage of the OD value of immunoreactive bands of empty pɛMTH vector-transfected control cells (normalized OD) regarded as 100% (line). In case of PKCε only the densitometric values are shown because of its undetectable expression in control cells. Points represent the mean ± SEM of three independent experiments. * marks significant (*P* < 0.05) differences compared to non-transfected control. The figures are representative of three experiments for each isoform yielding similar results. (B) Cell lysates of overexpressers and empty pɛMTH vector (Control, Co) transfected C2C12 cells were analysed for kinase activity by measuring ^32^P incorporation into Histone IIIS (H-III) or myosin light chain 20 (MLC20) substrates. The values are averages of three independent determinations and expressed as per cent of control (mean ± SEM) regarded as 100%.

For transfection, C2C12 or RD cells were seeded in 6-well tissue culture dishes and at 60–70% confluence and were transfected by either the empty pεMTH vector (control cells) or by the vectors encoding the cDNA sequences of PKCα, β, δ, ε or DN-nPKCδ 9,27,29,30. Transfections were performed with a Lipofectamine anionic detergent (Invitrogen) in serum-free DMEM solution using 2–4 μg cDNA according to the protocol suggested by the manufacturer. Cells were selected in DMEM containing 750 μg/ml G418 (Geneticin, Invitrogen) for 12–18 days, then single colonies were isolated. PKC overexpressing cells were cultured in supplemented DMEM containing 500 μg/ml G418. Experiments were routinely carried out on pools of transfected cells, but the results were confirmed on at least three individual clones for each isoform. The efficacy of recombinant overexpression was monitored by Western blotting and PKC kinase assays (see below and in Fig.1).

### Western blotting

Cells were homogenized in lysis buffer (20 mM Tris-Cl, 5 mM EGTA, pH 7.5 and protease inhibitor cocktail all from Sigma-Aldrich) disrupted by sonication on ice 9 and the protein content of samples was measured by the BCA protein assay kit (Pierce, Rockford, IL, USA). Total cell lysates were mixed with SDS-PAGE sample buffer and boiled for 10 min. at 100°C. The samples were subjected to SDS-PAGE (7.5% gels were loaded with 20 μg protein per lane) and transferred to nitrocellulose membranes (Bio-Rad, Wien, Austria). Membranes were then blocked with 5% dry milk in PBS and probed with the appropriate primary antibodies against the given PKC isoforms, differentiation marker desmin and ERK 1/2 or phosphor-ERK 1/2. Peroxidase-conjugated goat anti-rabbit or antimouse IgG antibodies (Bio-Rad) were used as secondary antibodies, and the immunoreactive bands were visualized by SuperSignal West Femto Chemiluminescent Substrate-enhanced chemiluminescence (Pierce) using a Gel Logic 1500 Imaging System (Kodak, Tokyo, Japan). Immunoblots were subjected to densitometric analysis using an Intelligent Dark Box (Fuji, Tokyo, Japan) and the Image Pro Plus 4.5.0 software (Media Cybernetics, Silver Spring, MD, USA), and then normalized densitometric values of the individual lanes of several independent experiments were determined. As endogenous loading controls, expression of β-actin was determined using the procedure as described above.

### PKC activity (kinase) assay

The PKC activity of transfected C2C12 cells was determined as described before 9,24. Briefly, cells were lysed in the lysis buffer described above and the kinase activity of the cell lysates was determined using Histone IIIS (H-III, from Sigma-Aldrich) or the 20 kD light chain of smooth muscle myosin (MLC20), isolated from turkey gizzard, as substrates. The assay mixture contained 20 mM TRIS-HCl (pH 7.5), 20 mM MgCl_2_, 1 mM CaCl_2_, 25 μM [γ-^32^P]-ATP (600–1000 cpm/pmol) and 0.2 mg/ml H-III or MLC20. The reaction was started by the addition of [γ-^32^P]-ATP (Izinta Ltd., Budapest, Hungary) and assays were incubated at 30°C. Aliqouts were spotted on P81 phosphocellulose paper and washed three times in 500 ml of 0.5% phosphoric acid, then with acetone. Incorporation of ^32^P into the proteins was determined by counting the dried P81 papers in a scintillation counter. Data represent triplicate determinations.

### Determination of cellular proliferation

Proliferation of C2C12 myoblast was measured by a colorimetric bromo-deoxyuridine (BrdU) assay kit (Boehringer Mannheim, Mannheim, Germany) and by analysing standard growth curves 9. In those BrdU assays where the effects of PKC acting agents were tested on cellular proliferation, cells were plated in 96-well multititre plates (5000 cells/well density) in quadruplicate and 4 hrs later were treated with different concentrations of the agents and further incubated for the time indicated. Cells were then incubated with 10 μM BrdU for 4 hrs, and the cellular incorporation of BrdU (as the indicator of cellular proliferation) was determined colorimetrically according to the manufacturer's protocol. When BrdU assays were employed to investigate growth properties of PKC transfectants, cells were seeded at a density of 1000 cells/well and the BrdU incorporation was determined after the indicated days of culture, as described above.

Proliferation of RD cells was determined by measuring the conversion of the tetrazolium salt MTT (Sigma-Aldrich) to formazan by mitochondrial dehydrogenases. Cells were plated in 96-well multititre plates (1000 cells per well density) in quadruplicates and were cultured for 1–4 days. Cells were then incubated with 0.5 mg/ml MTT for 2 hrs and the concentration of formazan crystals was determined colorimetrically according to the manufacturer's protocol 32.

To assess doubling times and maximal cell numbers of PKC overexpressers, 10^4^ cells/well were plated in 12-well plates in triplicate in complete DMEM. Fresh medium was added every other day, and the cells in triplicate were harvested by trypsinization as indicated (usually on a daily basis) and counted using a hemocytometer. In the determination of the average doubling time, the 24 hr timepoint was used as the starting point to avoid artefacts because of the initial lag period after plating 9,29,30. The following equation was used to calculate doubling time: τ = D/log_2_(*N*/*N*_0_) where τ is the doubling time, D is the number of days of culturing, *N* and *N*_0_ are the number of cells at the end and the beginning of the experiments, respectively. To determine the maximal cell density, cells were grown in 12-well plates to confluence and kept post-confluent for 3 additional days with daily medium changes and then counted as described above.

### Xenograft experiments

Severe combined immunodeficiency (SCID) mice were bred and maintained in the animal facility of the Department of Dermatology (University of Debrecen) in accordance with the animal-welfare ordinance. The studies were performed under the current regulations and standards of the Institutional Research Ethics Committee of the University of Debrecen, Hungary. Cells were harvested by trypsinization and washed twice with DPBS. Cell pellets [2 × 10^6^ viable cells (C2C12 cells) and 4 × 10^6^ viable cells (RD cells)] were re-suspended in culture medium and injected in a single subcutaneously site on the right flank of SCID mice (0.2 ml/injection) and observed over a period of 30 days 9. Animals were finally killed and the averaged three-dimensional size and histological characteristics of the developed tumours (five animals for each group) were analysed.

### Immunohistochemistry

The histological parameters were determined on formalin-fixed, paraffin-embedded, and haematoxylin-eosin-stained sections of the developed tumours 9. Immunohistochemical images were captured and digitalized using an RT Spot Colour CCD camera (Diagnostic Instruments Inc.) integrated on a Nikon Eclipse 600 fluorescence and light microscope (Nikon). Digitalized images were then analysed using Image J (NIH, Bethesda, MD, USA) image analysis software. The averaged number of cell divisions was measured by counting the number of nuclei showing clear signs of mitosis in ten individual visual fields at high magnification using a light microscope. Results obtained in each tumour of the same group were then averaged and the mean values were calculated.

In addition, to assess the number of proliferating cells, formalin-fixed, paraffin-embedded sections were immunostained against the nuclear marker Ki67 9 using a streptavidin-biotin-complex (SABC) three-step immunohistochemical technique (DAKO, Hamburg, Germany). First, the inhibition of endogenous peroxidase activity was performed with 3% H_2_O_2_ in 100% methanol (both from Sigma-Aldrich). Then, non-specific binding was blocked by 1% bovine serum albumin (Sigma-Aldrich) in PBS buffer (pH 7.5). After testing various concentrations of the anti-Ki-67 monoclonal mouse primary antibody (DAKO), an optimal 1:50 dilution was employed. The sections were then incubated in a humid chamber using a biotin-coupled antimouse secondary antibody (1:100, DAKO) followed by streptavidin conjugated with horseradish peroxidase (1:600, DAKO). To reveal the peroxidase activity, VIP SK-4600 (Vector, Burlingame, CA, USA) was employed as a chromogene. The tissue samples were finally slightly counterstained with methyl green (DAKO) and mounted with Aquatex (Merck, Wien, Austria). The averaged number of proliferating (Ki67 positive) cells was measured by counting the total number of Ki67 positive cells at five randomly placed, equal areas of interest and the values were normalized to the total number of cells measured at the fields.

### Statistical analysis

The data are expressed as mean ± SEM. Significance differences were assessed by a two-tailed un-paired *t-*test (*P* < 0.05 values were defined as significance).

## Results

### Overexpression of certain cPKC and nPKC isoforms in C2C12 myoblasts

In the initial phase of our experiments, using the previously introduced MTH vectors 9,29–31, we have stably transfected C2C12 myoblasts with cPKCα and β and nPKCδ and ε (similar to as we have shown before 24). We first examined the efficacy of recombinant overexpression. Cell lysates of pooled cultures were subjected to Western blotting employing isoform-specific antibodies that corresponded to the overexpressed recombinant PKC isoforms, we found that the levels of the overexpressed PKCs (Fig.1A) were several-fold higher than those of the respective endogenous ones. To establish that the overexpressed PKC isoforms were functionally active, we also measured kinase (PKC) activity in cell lysates. As seen in Figure1B, the cells expressing the recombinant PKC isoforms showed higher kinase activity, as assessed with both kinase substrates, compared with the control (empty vector-transfected) C2C12 cells.

### PKC isoforms differentially alter cellular proliferation and expression of the differentiation marker desmin in C2C12 myoblasts

**Fig 2:**
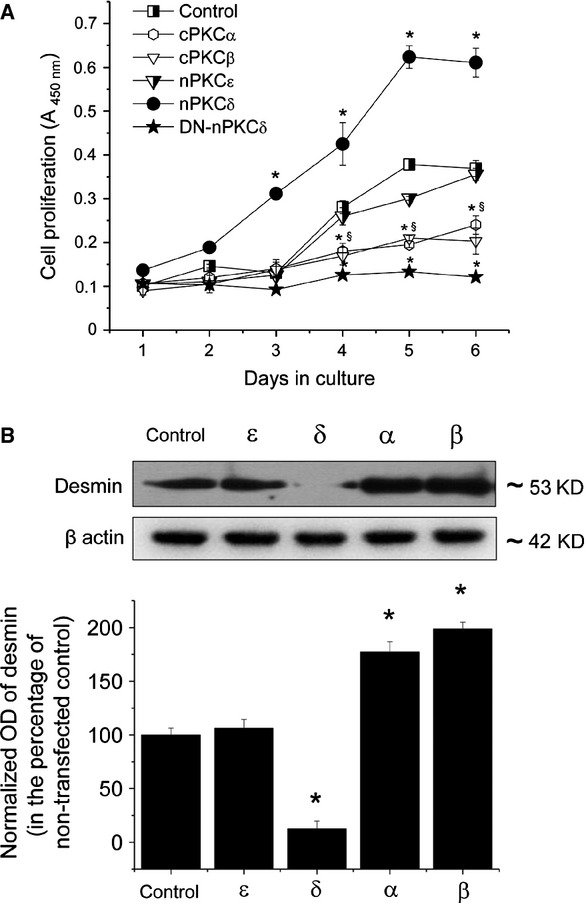
Effects of overexpression of PKC isoforms on the proliferation and differentiation of C2C12 myoblasts. (A) Control and PKC overexpresser C2C12 cells were seeded at densities of 1000 cells/well in 96-well microtitre plates and cell proliferation was determined after the indicated days of culture using BrdU assays. Points represent the mean ± SEM of quadruplicate determinations in one representative experiment for each isoform. At least three additional experiments for each PKC isoform yielded similar results. * marks significant (*P* < 0.05) differences compared to control in case of cPKCα. § marks significant (*P* < 0.05) differences compared to control in case of cPKCβ. (B) Stable transfectants of C2C12 cells overexpressing the different PKC isoforms or the control empty pɛMTH vector (Control) were harvested, similar amounts of proteins were subjected to SDS-PAGE, and the Western immunoblotting was performed with a mouse antibody against the differentiation marker desmin. To assess equal loading, membranes were re-probed with β-actin antibody (β actin). The amounts of desmin were quantitated by densitometry (optical density, OD), and expressed as the percentage of the OD value of immunoreactive bands of empty pɛMTH vector-transfected control cells (normalized OD) regarded as 100%. Points represent the mean ± SEM of three independent experiments. Three other experiments yielded similar results. * marks significant (*P* < 0.05) differences compared to control.

We then investigated the effect of overexpression of the PKC isoforms on the proliferation of C2C12 myoblasts. As revealed by BrdU assays (Fig.2A) and standard growth curve analyses (Table1), the overexpression of the PKC differentially affected the growth of the cells. The overexpression of cPKCα and β markedly decreased the proliferation of C2C12 cells whereas transfection with nPKCε resulted in insignificant changes in the growth rate. Conversely, myoblasts overexpressing the nPKCδ (confirming our previous finding 24) exhibited dramatically higher proliferation rates compared to the control (empty vector-transfected) cells.

**Table 1:** In vitro and *in vivo* growth analysis of C2C12 cells overexpressing various PKC isoforms

Isoform	*In vitro* growth analysis		*In vivo* tumour growth analysis		
	Doubling time (hrs)	Saturation density (10^5^ cells/cm^2^)	Averaged tumour size (mm)	Number of cell division	Percentage of Ki67 positive cells
Control	29.4 ± 3.6	1.2 ± 0.1	6 × 6×3.5	2 ± 0.3	18.2 ± 3.8
cPKCα	33.5 ± 3.8	1.1 ± 0.3	5 × 5×3	1.8 ± 0.3	15.2 ± 2.9
cPKCβ	31.8 ± 4.9	1.1 ± 0.1	4.5 × 5×3.5	1.5 ± 0.5	13.8 ± 4.6
nPKCε	28.5 ± 4.3	1.3 ± 0.2	6 × 5×3	2.2 ± 0.8	20.5 ± 5.9
*nPKCδ	11.6 ± 3.8	3.6 ± 0.5	23 × 18 × 11	25 ± 1.5	88.5 ± 6.2
*DN-nPKCδ	73.6 ± 5.9	Not measurable	Did not induce tumours		

* All data obtained with the nPKCδ and DN-nPKCδ are significantly (*P* < 0.05) different from those of the control cells (see text for further details).

Various parameters were analysed as described under ‘Materials and methods’. Data are expressed as mean ± SEM, except for averaged tumour size, where the three-dimensional sizes of three–four tumours per group were averaged and the mean values are shown.

Differences in proliferation were paralleled by the average doubling times and saturation densities of the cultures (Table1). Consistent with findings in the BrdU assays, cPKCα and β overexpressers possessed prolonged doubling times and decreased saturation densities, while cells that overexpressed nPKCδ were characterized by markedly increased saturation densities and shortened doubling times (again, nPKCε-transfectants showed similar growth properties when compared to those of the control C2C12 cells).

To follow differentiation, we measured the expression of the muscle-specific differentiation marker desmin in the PKC overexpressing cells. To obtain comparable data, all cell cultures were harvested at about 80-85% of confluence, equal amounts of protein were subjected to SDS-PAGE, and the expression of desmin was investigated by Western blotting. As seen in Figure2B, in cells overexpressing the cPKCα and β isoenzymes, the levels of the differentiation marker increased, whereas in myoblasts overexpressing the nPKCδ isoforms the levels of the desmin remarkably decreased compared to those of the control C2C12 cells (data obtained with nPKCε overexpressers, again, revealed no differences).

### PKC inhibitors differentially modify whereas the kinase-negative mutant of nPKCδ (DN-nPKCδ) inhibits cellular proliferation of C2C12 myoblasts

The above findings strongly suggested that in C2C12 myoblasts (*i*) the cPKCα and β isoforms inhibit proliferation and promote differentiation; (*ii*) the nPKCδ, in contrast, markedly stimulates cell growth but inhibits differentiation; and (*iii*) the nPKCε plays an insignificant role in regulating the above processes. To further investigate these proposals, we measured the effects of certain PKC inhibitors on proliferation of control C2C12 myoblasts. In addition, similar to as previously reported 24, we constructed such C2C12 myoblasts which stably overexpress the kinase-negative mutant of nPKCδ (DN-nPKCδ) and measured the effects of this recombinant modification on the cell growth of the cells.

**Fig 3:**
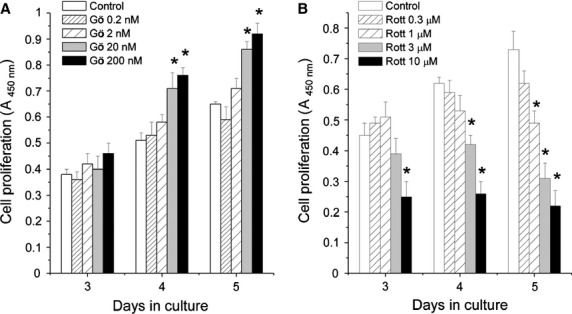
Effects of PKC inhibitors on cellular proliferation of C2C12 myoblasts. C2C12 cells were seeded at densities of 5000 cells/well in 96-well microtitre plates, treated with various concentrations of (A) Gö6976, inhibitors of the cPKCs or (B) Rottlerin, inhibitor of nPKCδ, and then BrdU assays were performed at certain culturing days. Points represent the mean ± SEM of quadruplicate determinations in one representative experiment. Two other experiments yielded similar results. * marks significant (*P* < 0.05) differences compared to the daily-matched untreated control.

As seen in Figure3, Gö6976, an inhibitor of the cPKC isoforms 33 (*i.e*. the cPKCα and β in C2C12 cells) stimulated the proliferation of the cells in a dose-dependent manner (Fig.3A). In contrast, the nPKCδ inhibitor Rottlerin 34 dose-dependently inhibited cellular growth (Fig.3B). Furthermore, confirming our previous results 24, the overexpression of DN-nPKCδ resulted in a dramatically suppressed cellular proliferation rate (Fig.2A) and prolonged doubling time (Table1; actually, cell cultures of DN-nPKCδ overexpressers never reached confluence; hence, the saturation density of these cultures was not measurable). Although confidence in the interpretation is limited because of possible effects of Gö6976 and Rottlerin on systems other than PKC 34,35, these findings may further argue for that cPKCα and β are negative while nPKCδ is indeed a positive regulator of proliferation in C2C12 myoblasts.

### Cells overexpressing nPKCδ induce malignantly transformed, large tumours in SCID mice

**Fig 4:**
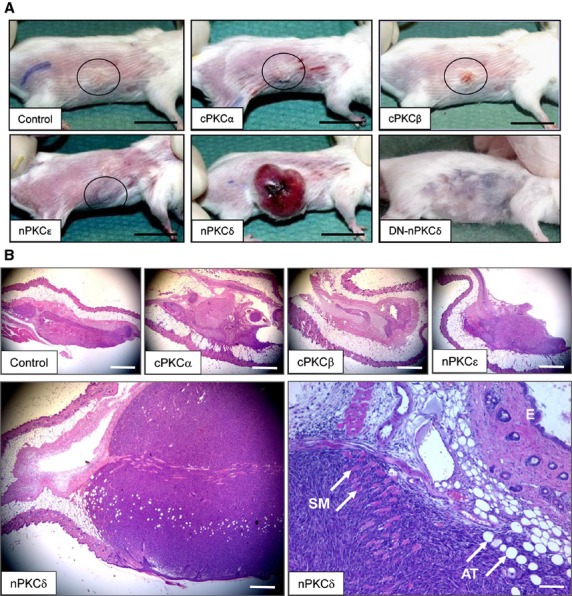
Effects of overexpression of PKC isoforms on tumourigenicity in SCID mice. Stable transfectants of C2C12 cells overexpressing the different PKC isoforms or the empty vector (Control) were injected subcutaneously to SCID mice. (A) Representative images of mice with tumours at day 30. Circles indicate tumours; scale bar: 20 mm. (B) After 30 days, animals were sacrificed, the developed tumours were excised and haematoxylin-eosin staining was performed on formalin-fixed paraffin-embedded sections. Lower right image: note that the aggressively growing tumour induced by nPKCδ overexpressers infiltrated and destroyed the subcutaneous adipose tissue (AT) and skeletal muscle (SM) layer (E, epidermis). Scale bar: 1 mm, except for the lower right image where scale bar indicates 200 μm.

We then investigated the behaviour of PKC overexpressing cells in assays for tumour formation and *in vivo* growth. SCID mice (four in each group) were injected with cell suspensions of C2C12 myoblasts overexpressing different PKC isoforms and, after 30 days, the developed tumours (Fig.4A) were characterized. As revealed on haematoxylin-eosin-stained sections, control (empty vector-transfected) C2C12 cells formed small tumours with expansive growth properties at the periphery and with signs of rhabdoid differentiation at the centre of the tumour (Fig.4B). The injection of C212 cells overexpressing cPKCα, β or nPKCε isoforms, when compared to the control ones, generally did not change the major histological characteristics of the tumours. Namely, these small tumours maintained the expansive (*i.e*. non-infiltrative, benign) growth characteristics and histological features of peripheral proliferation and rhabdoid differentiation. In addition, we found only minor differences in the average size of the tumours, the number of dividing cells, and the percentage of Ki67+ (hence proliferating) cells on the histological sections of tumours (these values were somewhat smaller in those tumours which were induced by cells overexpressing cPKCα and β, when compared to the control ones; Table1).

Of great importance, however, cells overexpressing nPKCδ induced the development of extremely large tumours (often with superficial exulceration and bleeding) which, in numerous cases, resulted in significant weight loss and eventually death of the animals within the 30-day investigation period (Fig.4A, Table1). Histologically, these tumours were characterized by markedly high cell division rate (as reflected by the elevated number of mitosis and Ki67+ cells), infiltrating (hence malignant) growth properties resulting in destruction of various layers of different cell types of the skin, and complete lack of rhabdoid differentiation (Fig.4B). Therefore, these tumours could be diagnosed as malignant RMSs. Finally, it was also important to observe that C2C12 myoblasts overexpressing the DN-nPKCδ failed to induce any tumour when injected intradermally to SCID mice (Table1).

### nPKCδ promotes cellular growth of human RMS cells

**Fig 5:**
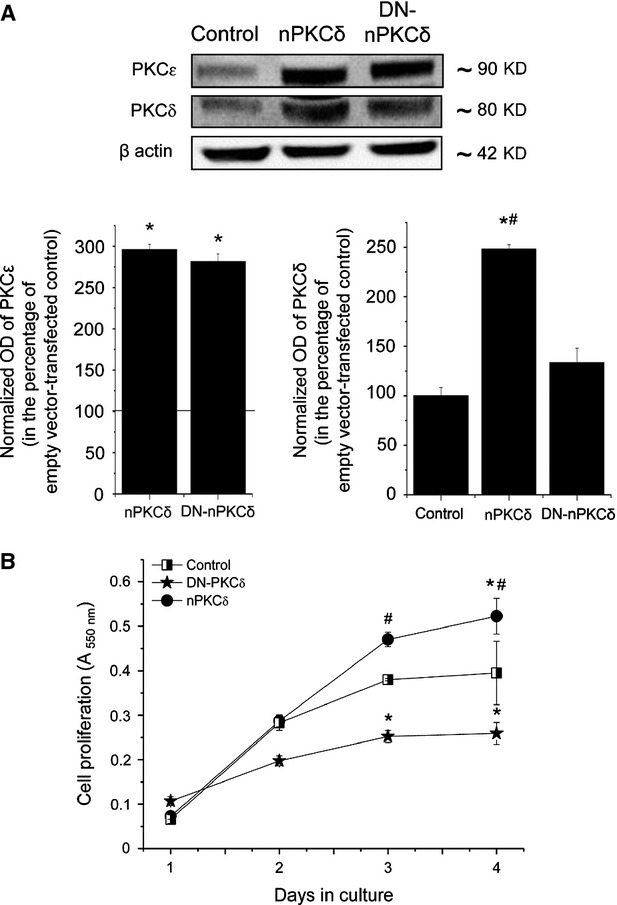
Effect of overexpression of nPKCδ on cellular proliferation of human rhabdomyosarcoma-derived RD cells. (A) Stable transfectants of RD cells overexpressing nPKCδ or the dominant-negative (DN-nPKCδ) nPKCδ mutant or the empty vector (Control) were harvested, similar amounts of proteins were subjected to SDS-PAGE, and the Western immunoblotting was performed with antibodies recognizing nPKCδ isoform or ε-tag. To assess equal loading, membranes were re-probed with β-actin antibody (β actin). The amounts of nPKCδ isoform were quantitated by densitometry (optical density, OD), and expressed as the percentage of the OD value of immunoreactive bands of empty pɛMTH vector-transfected control cells (normalized OD) regarded as 100%. Points represent the mean ± SEM of three independent experiments. * marks significant (*P* < 0.05) differences compared to pɛMTH control, while # marks significant (*P* < 0.05) differences compared to DN-nPKCδ mutant overexpressing cells. The figure is a representative of two experiments yielding similar results. (B) Control, nPKCδ and DN-nPKCδ overexpresser RD cells were seeded at densities of 1000 cells/well in 96-well microtitre plates and cell proliferation was determined after the indicated days of culture using MTT assay. Points represent the mean ± SEM of quadruplicate determinations in one representative experiment for each mutant. * marks significant (*P* < 0.05) differences compared to control.

The above data strongly argued for the key role of nPKCδ in promoting proliferation and inducing malignant transformation of myoblasts. To further assess these phenomena, in the next steps of our experiments, we have stably transfected human RMS-derived RD cells either with empty MTH (pεMTH) vector or with vectors encoding the active (nPKCδ) or dominant-negative (DN-nPKCδ) isoform. Similar to as described in Figure1, Western blot analyses revealed that the levels of the overexpressed nPKCδ was several-fold higher compared to the control (empty vector expressing) cells (Fig.5A). nPKCδ antibody is unable to differentiate between the endogenous and the ectopically overexpressed nPKCδ isoform and it also recognizes DN-nPKCδ. Therefore, to make the differentiation possible, we have performed another Western blot analysis, but now using an anti-PKCε antibody which, besides the endogenous PKCε, also recognizes the ε-tag of the recombinantly overexpressed nPKCδ and DN-nPKCδ. As seen in Figure5A, the densitometry analysis of the various immunoreactive bands revealed that whereas the PKCε-specific signals of the nPKCδ and the DN-nPKCδ samples were very similar and significantly stronger that measured in the control samples, the nPKCδ-specific immunoreactivity was significantly higher only in the PKCδ but not in the DN-nPKCδ group. These results strongly suggest that, upon DN-nPKCδ transfection and overexpression, the level of the endogenous nPKCδ was markedly suppressed.

We then investigated the effect of overexpression of the nPKCδ mutants on the *in vitro* proliferation of RD cells. As revealed by growth curve analysis (Fig.5B), the overexpression of nPKCδ significantly increased the proliferation of RD cells compared to control (empty vector-transfected) cells. Further, DN-nPKCδ overexpresser RD cells exhibited a significantly suppressed growth rate when compared to control.

### nPKCδ is involved in IGF-I-induced ERK 1/2 activation in RMS cells

**Fig 6:**
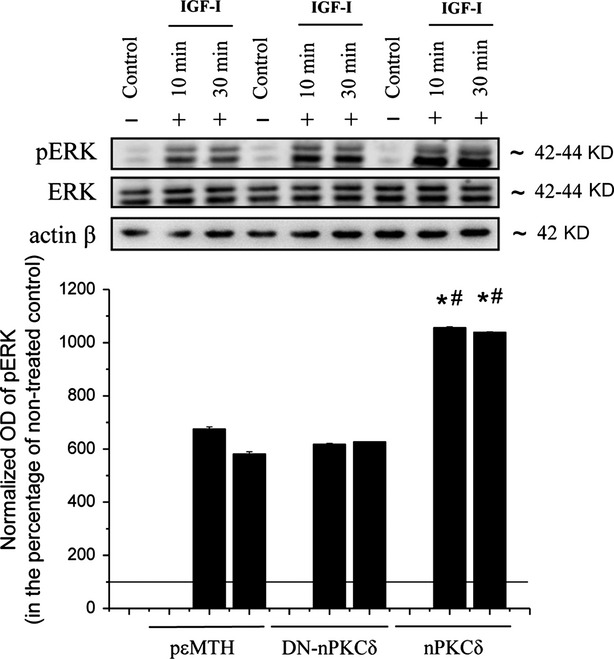
Effect of overexpression of nPKCδ on the Ras-related signalling of RD cells. nPKCδ and DN-nPKCδ overexpresser RD cells, as well as controls, were treated with IGF-I (100 ng/ml) as indicated. ERK 1/2 and phosphorylated ERK 1/2 were detected by immunoblotting of cell lysates. To assess equal loading, membranes were re-probed with β-actin antibody (actin β). The amounts of pERK 1/2 were quantitated by densitometry (optical density, OD), and expressed as the percentage of the OD value of immunoreactive bands of non-treated control cells (normalized OD) regarded as 100% (line). Points represent the mean ± SEM of two independent experiments. *marks significant (*P* < 0.05) differences compared to empty pɛMTH vector-transfected control, while # marks significant (*P* < 0.05) differences compared to DN-nPKCδ mutant overexpressing cells.

Insulin-like growth factor-I is reported to be a significant growth factor in skeletal muscle biology and physiology 24,36–38. To uncover the potential mechanism by which nPKCδ modulates the proliferation and tumourigenicity of RMS cells, we also evaluated the role of nPKCδ in modulating the IGF-I induced activation of the Ras-MAPK signalling pathway. Cells were treated with IGF-I (Fig.6) as indicated, and the activation of the possibly most important downstream molecule related to Ras, ERK 1/2 kinase, was examined by Western blot. We show that the overexpression of nPKCδ enhanced the IGF-I-induced ERK 1/2 phosphorylation (Fig.6) compared to the cells overexpressing the dominant-negative mutant (DN-nPKCδ) or the empty vector (control) suggesting the involvement of nPKCδ in mediating the growth-promoting effect of IGF-I (similar to as we have previously shown for human skeletal muscle cells and C2C12 myoblasts 24).

### Role of nPKCδ on tumourigenesis of human RD cells

**Fig 7:**
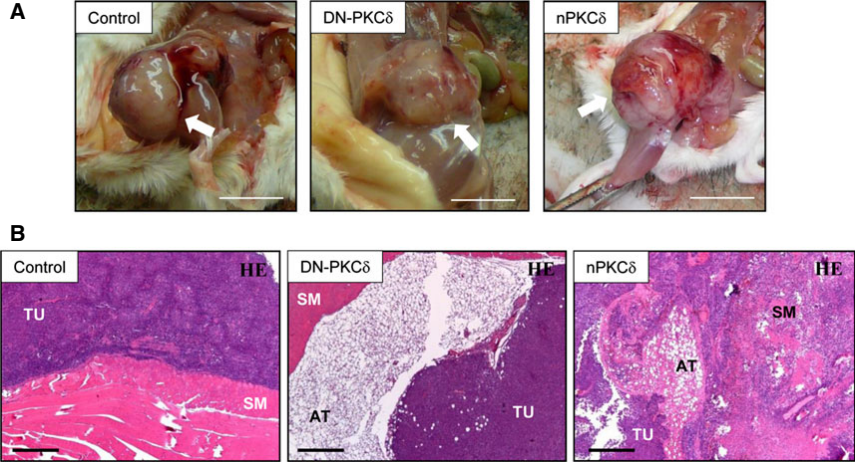
Effect of overexpression of nPKCδ and DN-nPKCδ on tumourigenicity of rhabdomyosarcoma cells. (A) Representative images of tumours (indicated by arrows) induced by nPKCδ, DN-PKCδ or empty vector-transfected RD cells at day 30; scale bar: 10 mm. (B) After 30 days, animals were killed, the developed tumours were excised and haematoxylin-eosin staining was performed on formalin-fixed paraffin-embedded sections. Note that the tumour (TU) induced by nPKCδ overexpresser RD cells infiltrated and destroyed the subcutaneous adipose tissue (AT) and skeletal muscle (SM) layers; scale bar: 100 μm.

To establish the relevance of nPKCδ in RMS tumourigenesis, we also investigated the role of the nPKCδ isoform in the *in vivo* tumour formation of RD cells. For this, tumours were induced in SCID mice (five in each group) by injecting RD cells overexpressing either nPKCδ, DN-nPKCδ or the empty vector (control). As expected, injection of all RMS-derived RD cell types resulted in tumour development in immunodeficient mice (Fig.7A). Histologically, these tumours were diagnosed as malignant RMSs with high cell division rates (number of mitosis) and infiltrating (malignant) growth properties, very often destructing the neighbouring adipose and skeletal muscle tissues (Fig.7B and Table2). Among them, tumours induced by nPKCδ overexpressers were characterized by the largest three-dimensional size and the highest percentage of Ki67 positive (*i.e*. proliferating) cells within the sarcomas; the latter value was significantly different from those measured in tumours induced by control or DN-nPKCδ overexpressing cells. Interestingly, features of tumours induced by DN-nPKCδ overexpressers did not significantly differ from those of the control RD cells. These differential features of the various cells on tumourigenesis were also proven by immunohistochemical analysis of the expression of the proliferation marker Ki67.

**Table 2:** *In vivo* growth analysis of RD cells overexpressing nPKCδ or DN-nPKCδ

Isoform	*In vivo* tumour growth analysis		
	Averaged tumour size (mm)	Number of cell division	Percentage of Ki67 positive cells
Control	22 × 14.6 × 12.6	14.6 ± 3.0	82.75 ± 8.13
nPKCδ	25 × 18 × 15.2	16.6 ± 1.5	97.6 ± 4.32*
DN-nPKCδ	21.25 × 16 × 12	17.1 ± 4.4	78.31 ± 7.28

* Data obtained with the nPKCδ is significantly (*P* < 0.05) different from those of the control and DN-PKCδ cells (see text for further details).

Various parameters were analysed as described under ‘Materials and methods’. Data are expressed as mean ± SEM, except for averaged tumour size, where the three-dimensional sizes of five tumours per group were averaged and the mean values are shown.

## Discussion

In this study, we provide the first evidence that certain cPKC and nPKC isoforms play differential and antagonistic roles in regulating the *in vitro* proliferation and differentiation of C2C12 myoblasts as well as *in vivo* tumour growth induced by these cells. Using molecular biological (recombinant overexpression) methods combined with pharmacological modifications (inhibition) of the PKC isoform activities, we have shown that the ‘conventional’ cPKCα and β act as negative regulators of cellular growth and, moreover, their activities stimulate differentiation of the cells (Figs2 and 3). Interestingly, in cultured avian myoblasts, Capiati *et al*. 18,19 have elegantly proven that cPKCα played a central in promoting cellular growth which findings contradict with our current data. Moreover, we have previously reported that this isoform did not participate in the growth-inhibitory action of the PKC activator phorbol esters in human cultured skeletal muscle cells 28. These data, therefore, suggest that the regulatory role of cPKCα to affect skeletal muscle proliferation possesses marked species dependence.

The nPKCε has been extensively documented as a key molecule to promote cellular proliferation in various cell types 3,5–7. It was also shown that the overexpression of nPKCε increased *in vivo* and *in vitro* cellular growth whereas its down-regulation resulted in inhibition of proliferation and induction of differentiation 5,7–9. In the current study, however, we found that this isoform plays an insignificant role in regulating proliferation, differentiation, and the tumour inducing properties of C2C12 myoblast (Figs2 and 4, Table1). As we and others have failed to identify this isoform in C2C12 cells 24, which finding was also confirmed in this study (Fig.1A), the lack of effect of recombinant overexpression of the constitutively active (Fig.1B) nPKCε on cellular growth of the myoblasts is most probably because of the lack of the signalling – substrate system related to this isoenzyme.

Our most remarkable data in this investigation were obtained with nPKCδ. This isoform was also very often implicated in the regulation of cellular proliferation and differentiation of numerous cell types 3,5–8. However, in most studies (for example, in human keratinocytes 9,31 and fibroblast 29,30) the isoform was suggested to stimulate differentiation and apoptosis and to inhibit proliferation, whereas, up to the start of the current study, nPKCδ was shown to stimulate proliferation (acting as a prosurvival factor) only in certain breast cancer cell lines 39.

Of great importance, our current findings introduce nPKCδ as a novel significant player in skeletal muscle biology positively controlling cellular growth. These statements were supported by the following data: (*i*) overexpression of the constitutively active nPKCδ stimulated whereas the kinase inactive DN-nPKCδ mutant inhibited *in vitro* growth of C2C12 myoblasts (Fig.2A); (*ii*) overexpression of nPKCδ suppressed the expression of the differentiation marker desmin (Fig.2B); (*iii*) the inhibition of PKCδ activity by Rottlerin inhibited cellular proliferation of the control C2C12 cells (Fig.3B); (*iv*) nPKCδ overexpresser C2C12 cells, when injected to immunodeficient mice, initiated the development of large and, of great importance, malignantly transformed RMSs (in contrast to control myoblasts which induced benign tumours of much smaller size) (Fig.4 and Table1); and (*v*) DN-nPKCδ overexpresser myoblasts did not induce tumours in SCID mice. Moreover, the above argument is also supported by our previous report presenting that nPKCδ plays a central role in mediating the mitogenic effect of IGF-I, one of the key autocrine – paracrine growth factors in skeletal muscle physiology and pathology 40, both in human and C2C12 skeletal muscle cells 24.

Protein kinase C isoforms have been implicated in the pathogenesis of numerous human malignancies including breast, colon, lung, prostate, pancreatic, liver and hematopoietic ones 41,42. RMS is a group of aggressive muscle tumours and the most common soft tissue sarcomas in children 43. The poor clinical outcomes foster trials for a better understanding of the tumourigenic mechanisms so that new therapeutic targets can be identified 44,45. Although only few reports are available on describing the expression profile of the PKC family in RMS, involvement of individual PKC isoforms and their use as therapeutic targets are beginning to be explored 46–48. As we found that C2C12 myoblasts overexpressing nPKCδ induced malignant tumours in immunodeficient mice (Fig.4), we sought to define the exact functional role of this isoform in RMS tumourigenesis. Importantly, overexpression of nPKCδ further enhanced the already highly accelerated cell proliferation of human RMS-derived RD cells, compared to control cells or DN-nPKCδ overexpressers (Fig.5B).

Insulin-like growth factor-I is known as a potent mitogenic factor for RMS, expressions of IGF-I receptor have reportedly been elevated in the disease 49–51. Although we have previously identified that nPKCδ is involved in the IGF-I induced ERK 1/2 activation in C2C12 24, here we provide the first evidence that nPKCδ also contributes to signalling downstream of IGF-I in RD cells by modifying the level of IGF-I induced phosphorylation of ERK 1/2. Indeed, overexpression of nPKCδ increased the activation of ERK 1/2 induced by IGF-I stimulation compared to the pεMTH vector or DN-nPKCδ overexpresser cells (Fig.6).

Furthermore, overexpression of nPKCδ further increased xenograft tumour growth as well as the proliferation rate of the developed tumours (Ki67 positivity; Fig.7 and Table2). Interestingly, the size of tumours induced by cells overexpressing the inactive DN-nPKCδ did not differ from the control tumours suggesting that other factors than nPKCδ may also be involved in promoting the aggressive growth of RMS-derived cells. Nevertheless, these data (again) strongly suggest that nPKCδ may play a central role in RMS tumourigenesis.

Comparison of the current data with our previous experimental findings 9 revealed another intriguing phenomenon. In human epidermal keratinocytes, using identical molecular biological and pharmacological methods, we found that the overexpression of cPKCα and nPKCδ stimulated cellular differentiation and inhibited cellular proliferation and tumour growth. Conversely, the activity of cPKCβ and nPKCε increased both *in vitro* and *in vivo* growth of cells and inhibited differentiation. As our current investigation on skeletal muscle cells resulted mostly opposite findings (cPKCβ inhibited growth, nPKCε played minor role in the regulation of proliferation, nPKCδ markedly enhanced cellular and tumour growth), these data strongly suggest that certain PKCs not only isoform-specifically regulate cellular proliferation and differentiation but their effect exert a marked cell-type dependence as well.

In conclusion, in this study we present the first evidence that certain cPKC and nPKC isoforms play specific, yet antagonistic roles in regulating the *in vitro* and *in vivo* growth of C2C12 muscle cells. In addition, we describe nPKCδ as a novel key player in promoting cellular growth and inducing malignant transformation, which findings introduce this isoform as a promising, therapeutically exploitable, novel target for the treatment of skeletal muscle malignancies.
